# HbA1c Screening for Diabetes in Patients with Acute Coronary Syndrome: A Worthwhile Test or a Pitfall?

**DOI:** 10.3390/jcm10194334

**Published:** 2021-09-23

**Authors:** Robert Józwa, Marta Bryśkiewicz, Krzysztof Safranow, Liliana Majkowska

**Affiliations:** 1 Department of Cardiology and Invasive Cardiology, Provincial Hospital, ul. Arkońska 4, 71-455 Szczecin, Poland; rjozwa@outlook.com; 2 Department of Diabetology and Internal Diseases, Pomeranian Medical University, ul. Siedlecka 2, Police, 72-010 Szczecin, Poland; martab@pum.edu.pl; 3 Department of Biochemistry and Medical Chemistry, Pomeranian Medical University in Szczecin, Powstańców Wielkopolskich Av. 72, 70-111 Szczecin, Poland; chrissaf@mp.pl

**Keywords:** acute coronary syndrome, diabetes mellitus, undiagnosed glucose metabolism abnormalities, HbA1c, oral glucose tolerance test

## Abstract

Background: Diagnostic concordance between HbA1c and other glucose-based tests is imperfect, and data on this problem in acute coronary syndrome (ACS) are still lacking. The aim of this study was to identify undiagnosed glucose abnormalities in ACS patients, and to compare the effectiveness and consistency of the diagnostic screening based on HbA1c to the oral glucose tolerance test (OGTT). Methods: The study group consisted of 121 ACS patients, mean age 62.3 ± 11.6 years, without known glucose abnormalities. HbA1c, admission and fasting plasma glucose in the first days of hospitalization were assessed and referred to the results of OGTT performed two weeks after discharge. Results: OGTT identified normoglycemia in 45%, pre-diabetes in 39.4%, and diabetes in 15.6%, while HbA1c revealed these categories in 39.7%, 51.2%, and 9.1%, respectively. With an HbA1c cut-off ≥6.5% (48 mmol/mol) diagnostic for diabetes, the sensitivity of the method was 41%, while specificity was 98%, compared to the OGTT. The optimal HbA1c cut-off value at the crossing of sensitivity and specificity curves was 5.9%. The HbA1c value recommended for the diagnosis of pre-diabetes and optimal cut-off point were the same (5.7%). Conclusions: Using HbA1c without OGTT in an early but stable phase of ACS may result in a significant underdiagnosis of diabetes.

## 1. Introduction

The majority of patients with coronary artery disease may have undiagnosed abnormalities of the glucose metabolism [1,2,3]. Four criteria are recommended in the diagnosis of diabetes or prediabetes: fasting plasma glucose (FPG), 2-h plasma glucose (2-h PG) in an oral glucose tolerance test (OGTT), glycated hemoglobin HbA1c, or a random glucose test in a patient with classic symptoms of hyperglycemia [4,5,6,7]. According to the standards of medical care in diabetes FPG, 2-h PG during 75-g OGTT, and HbA1c are equally appropriate for diagnostic screening [7]. The same standards, however, stress that concordance between FPG and 2-h PG tests is imperfect, as is the concordance between HbA1c and either glucose-based test. It was shown that HbA1c, at the designated cut-off point of ≥6.5% (48 mmol/mol), diagnosed only 30% of the diabetes cases that were collectively identified using HbA1c, FPG, or 2-h PG [8]. Recently published studies have shown that 2-h PG values diagnose more people with diabetes and prediabetes than FPG and HbA1c [9,10,11]. The mismatches in the HbA1c v. glucose levels may impact the diagnostic classification of diabetes and prediabetes, and, therefore, FPG and 2-h PG have been indicated to be more accurate than HbA1c values in the diagnostic procedure [10]. The EUROASPIRE IV and Euro Heart Survey on Diabetes and the Heart found similar data in patients with coronary artery diseases; the OGTT diagnosed a greater proportion as having glucose abnormalities than FPG or HbA1c [3,12].

Despite its great diagnostic importance, 2-h PG values are rarely assessed in clinical practice, as OGTT is burdensome, costly and cannot be performed over the course of acute illness. In real clinical settings, HbA1c usage in the diagnostic procedures of glucose abnormalities is increasing, both in primary care and at admission to the hospital. HbA1c has several advantages over FPG or an OGTT in acute settings. Fasting is not required for the HbA1c test, preanalytical stability is much better than for glucose, the test reflects average glycemia over the preceding 2–3 months, and day-to-day changes are not significant. The test is not affected by stress-induced changes in blood glucose levels (stress hyperglycemia) and may be used in acute clinical situations.

The current guidelines of the European Society of Cardiology (ESC), developed in collaboration with the European Association for the Study of Diabetes (EASD), recommend that the diagnosis of diabetes should be based on HbA1c or FPG, and on OGTT if still in doubt; however, it is stressed that, in acute coronary syndrome (ACS), OGTT should not be performer earlier than 4–5 days, to minimize false-positive results [13]. A recently published study of newly diagnosed diabetes after acute myocardial infarction largely based the diagnosis on a single measurement of HbA1c at baseline or at 1-month follow-up [14]. Despite the recommendations of ECS, and the convenience of using HbA1c in clinical practice, it is not clear which of the tests is most appropriate for detecting undiagnosed diabetes in subjects with ACS. Some studies indicate that HbA1c may be a useful screening tool in ACS patients [15,16]. To date, only one study has compared different diagnostic criteria to detect undiagnosed diabetes in patients with ACS, which showed that not performing an OGTT resulted in significant underdiagnosis of diabetes [17]. However, it is not clear whether this concerns all patients with ACS, as the study was conducted in patients with ACS with concomitant hyperglycemia at admission.

The aim of this study was to identify the occurrence of previously undiagnosed diabetes or prediabetes in patients with ACS, and to compare the effectiveness of diagnostic screenings based on HbA1c levels in comparison to complete OGTT performed shortly after discharge.

## 2. Material and Methods

This study was conducted at a single center (Dept. of Cardiology and Invasive Cardiology, Provincial Hospital in Szczecin, Poland) over a 6-month period. The study group consisted of patients who were admitted to the coronary unit with a diagnosis of ACS, without previously known diabetes or other glucose abnormalities. Patients were enrolled into the study after standard ACS treatment if they gave consent for the OGTT procedure 2 weeks after discharge. Patients who denied consent, or had other medical conditions that could influence HbA1c measurements, such as kidney disease, anemia, iron or vitamin B12 deficiency or supplementation, were excluded. No tested patients were known to have haemoglobinopathies or conditions that affect erythrocyte lifespan. Subjects who declared problems with coming for OGTT visits in a proper term, mainly due to their living far from the city, were also excluded. Participants were enrolled after being provided with full information regarding the purpose and protocol of the study, and all subjects provided written informed consent to participate in the study. The study was approved by the Bioethics Committee of the Pomeranian Medical University in Szczecin (KB–0012/140/10) and conducted in accordance with the Declaration of Helsinki [18].

The studied group consisted of 121 patients of Caucasian ethnicity, with a diagnosis of ACS and without previous diagnosis of diabetes or prediabetes. Demographic, clinical and medical data were obtained for each patient, including age, sex, ethnicity, body mass, body mass index (BMI), blood pressure, history of hypertension, dyslipidemia, history of previous myocardial infarction and percutaneous coronary intervention (PCI) or coronary artery bypass graft (CABG). The diagnosis of ACS was based on the following criteria: typical ischemic chest pain or other clinical signs, typical changes in 12-leads ECG, and/or elevated markers of myocardial necrosis, i.e., increase in cardiac troponin T levels, which were measured on admission and within a 24-h period of the primary clinical event. The final ACS diagnosis was recorded as ST-elevation myocardial infarction (STEMI) or non-ST elevation myocardial infarction (NSTEMI). Coronary angiography was performed in 120 patients by radial or femoral routes; in one subject, angiography was not completed due to technical problems, and the patient was treated with thrombolysis (alteplase). Before angiography, all patients were given aspirin (300 mg), clopidogrel (600 mg) and 5000 IU of heparin. The assessment of the coronary artery disease was based on coronary angiograms, and was carried out by two experienced senior cardiologists. Subsequent management included medical therapy, PCI or referral for urgent CABG. The clinical characteristics of investigated subjects are shown in Table 1.

In the majority of patients (>50%) the burden of coronary disease was found for three arteries. The involvement of three coronary arteries was significantly more common in men than women (*p* < 0.01).

At admission, venous blood samples were taken for glucose, cardiac troponin T, creatinine, complete blood count, sodium and potassium ions, C-reactive protein (CRP), uric acid. Measurements of troponin T levels were repeated in subsequent hours according to ACS diagnosis standards. Estimated glomerular filtration rate (e-GFR) was calculated according to the MDRD formula. In the first two days of hospitalization, blood samples were taken fasting for glucose, HbA1c, and lipids (the total cholesterol, low-density lipoprotein cholesterol (LDL), high-density lipoprotein cholesterol (HDL), triglycerides). In a stable phase of the disease, approximately two weeks after discharge from the hospital, the OGTT with 75 g glucose load was performed in the morning, after an at least 8 h overnight fast, measuring fasting glucose levels and at the 2nd hour of the test. For safety reasons, a post-load glucose was only obtained when the FPG was <126 mg/dL (<7 mmol/L). OGTT was performed in 109 subjects, as 12 patients did not come for the test.

A biochemical analyses of all the parameters except HbA1c were carried out by the central hospital laboratory. The blood samples for glucose levels were collected into a container with glycolytic inhibitor and placed in ice-water until being separated prior to analysis. The glucose was assayed by the colorimetric method with hexokinase (Cobas 6000 Analyzer, Hitachi High Technologies Corporation, Tokyo, Japan). HbA1c levels were assessed using an NGSP-approved HPLC method (Variant Analyzer, Bio-Rad Laboratories, Hercules, CA, USA) at the laboratory of the Dept. of Diabetology and Internal Diseases of Pomeranian Medical University.

Glycemic status was classified on the basis of WHO/ADA criteria [5,6,7]. The OGTT criteria were used as the “gold standard”. Normal glucose metabolism was defined by FPG < 100 mg/dL (5.6 mmol/L) and 2-h PG in OGTT < 140 mg/dL (7.8 mmol/L), prediabetes as impaired fasting glucose (IFG) with FPG 100–125 mg/dL (5.6–6.9 mmol/L) and/or impaired glucose tolerance (IGT) with 2-h PG in OGTT 140–199 mg/dL (7.8–11.0 mmol/L), and diabetes by FPG ≥ 126 mg/dL (7.0 mmol/L) or 2-h PG in OGTT ≥ 200 mg/dL (11.1 mmol/L). HbA1c-based classification included normal values <5.7% (39 mmol/mol), prediabetes 5.7–6.4% (39–47 mmol/mol), and diabetes for values of ≥6.5% (48 mmol/mol).

HbA1c values, admission PG, and FPG in the first two days of hospitalization were assessed and referred to the results of OGTT performed after two weeks after discharge. Receiver operating characteristic (ROC) curves and the area under the curve (AUC) were calculated for HbA1c, using the OGTT as a gold standard, to study the diagnostic efficacy of the cut-off point for the diagnosis of diabetes (patients with diabetes vs. patients without diabetes, including those with prediabetes) and for the diagnosis of prediabetes (patients with prediabetes vs. patients with normoglycemia, after exclusion of patients with diabetes). The sensitivity and specificity of the cut-off HbA1c values currently used as the diagnostic standards for diabetes and prediabetes, and the optimal cut-off points for HbA1c at a sensitivity equal to the specificity, were assessed.

The laboratory parameters of investigated subjects are shown in Table 2.

### Statistical Analysis

Categorical data are presented as numbers and proportions, and continuous data are presented as the arithmetic mean ± standard deviation (SD) or as the median (lower quartile and upper quartile) for variables with an SD exceeding the mean value. Differences in the assessed parameters were evaluated by the chi^2^ test or Fisher exact test (dichotomous data). Since the distributions of most of the quantitative variables were significantly different from the normal distribution (Shapiro–Wilk test), non-parametric Mann–Whitney U test was used for comparison between independent groups. A *p* value < 0.05 was considered statistically significant. The statistical analysis was performed using STATISTICA version 13 with Plus bundle for ROC analysis (StatSoft, Kraków, Poland).

## 3. Results

The results of HbA1c, admission PG, FPG measured at the first two days of hospitalization, FPG and 2-h PG after glucose load during OGTT, performed two weeks after discharge, are presented in Table 3.

As the groups of men and women were not different with respect to age, BMI, blood pressure, medical history and laboratory parameters, which may impact the glucose metabolism, further analysis was performed for the total group of ACS patients.

The OGTT diagnostics two weeks after discharge were carried out for 109 patients (28 women and 81 men). The prevalence of different types of glucose abnormalities found in OGTT (diabetes, prediabetes defined as IFG and/or IGT), and the values of HbA1c, admission glycemia, and fasting glycemia in the hospital observed in these subgroups are presented in Table 4. 

Normal values of glycemia were found in 45% of 109 patients in whom OGTT was performed after a discharge. Pre-diabetes was diagnosed in 39.4% of subjects, with IFG in 18.3%, and IGT in 21.1%. The IGT group consisted of 13.8% subjects with both IFG and increased 2 h OGTT values, and 7.3% of subjects with an isolated increase in post-load glucose at 2 h of OGTT. Diabetes was found in 15.6%—i.e., in 17 of 109 subjects diagnosed after discharge. This group consisted of 11 subjects with fasting glycemia ≥126 mg/dL (7.0 mmol/mol)—in these cases, OGTT was not performed, and six subjects diagnosed by 2-h OGTT glucose values.

The HbA1c levels in the diabetes group were significantly higher than in other groups. The HbA1c levels in both subgroups of prediabetes (IFG and IGT) were significantly higher than in the normoglycemic group. The admission glycemia in subjects with diagnosis of diabetes was higher than in other groups (*p* < 0.01); however, the mean value of 166 ± 55 mg/dL (9.2 ± 3.1 mmol/L) was lower than the random glycemia indicative of diabetes. The admission glycemia in the normal, IFG and IGT groups were very similar.

In the diabetes group, the mean FPG at the first days of hospitalization was 135 ± 26 mg/dL (7.5 ± 1.4 mmol/L); this value was significantly higher compared to other groups (*p* < 0.01). The hospital FPG values in prediabetes subgroups (IFG and IGT) were very similar, but the IFG group had significantly higher values than normal-glycemic subjects (*p* < 0.04).

The percentage of different glucose abnormalities, suggested by FPG assessed at first two days of hospitalization, was very similar to the results shown by fasting and post-load OGTT results after discharge (Table 5). The admission glycemia was measured in 116 ACS patients. Random glucose levels suggesting diabetes (≥200 mg/dL; ≥11.1 mmol/L) were found in only five subjects, i.e., in 4.3%. Due to the very low number of these patients, further analysis of this group was not performed.

No severe glucose abnormalities were observed during hospitalization. The treatment (if needed) and lifestyle modification typical for diabetes or prediabetes were introduced after the OGTT visit.

The prevalence of diabetes and prediabetes in ACS patients based on different diagnostic methods is presented in Table 5.

Diagnostics based on HbA1_c_ levels revealed a lower number of diabetes and higher number of prediabetes than the procedure based on FPG and 2-h PG of OGTT, performed in ACS patients after discharge (*p* < 0.01) (Table 5). A direct comparison of classifications of glucose abnormalities based on HbA1c levels in the hospital and those based on the OGTT results, regarded as the reference method, is shown in Table 6. In the group of 17 patients with diabetes diagnosed during OGTT, HbA1c values indicative for diabetes were found in only seven subjects. In the group of 43 patients with prediabetes in OGTT (IFG and IGT) diagnosis of prediabetes based on HbA1c levels was found in 27 subjects, while two were in the diabetic range. 

**Figure 1:**
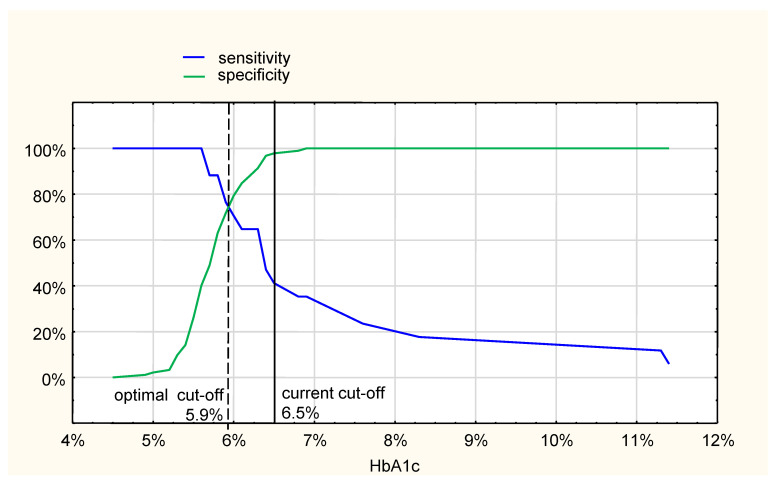
Sensitivity and specificity curves for HbA1c as a diagnostic method for diabetes compared with oral glucose tolerance test (OGTT), performed two weeks after discharge.

With HbA1c ≥ 6.5% (48 mmol/mol) recommended as a cut-off value diagnostic for diabetes, compared to the OGTT after discharge, the sensitivity was only 41%, while the specificity was 98% (Figure 1). The optimal cut-off HbA1c value, compared to OGTT, at the crossing of sensitivity and specificity curves, was 5.9%; however, the sensitivity and specificity were 77%, and 72%, respectively (Figure 1). The area under the ROC curve (AUC) was 0.86 (95%CI: 0.76–0.95).

**Figure 2:**
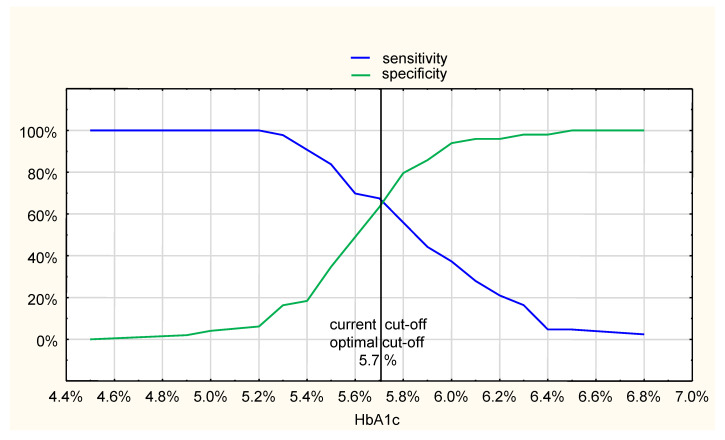
Sensitivity and specificity curves for HbA1c as a diagnostic method for pre-diabetes (IFG and IGT) compared with the oral glucose tolerance test (OGTT) performed two weeks after discharge.

Analysis of the HbA1c value that is diagnostic for prediabetes state (IFG and IGT after exclusion of diabetic patients), compared to the OGTT results, revealed that the cut-off point of 5.7% recommended by current diabetic care standards was the same as the optimal cut-off point, at the crossing of sensitivity and specificity curves, with a sensitivity of 67% and specificity of 63%, respectively (Figure 2). The AUC of ROC curve was 0.71 (95% CI: 0.60–0.81).

**Figure 3:**
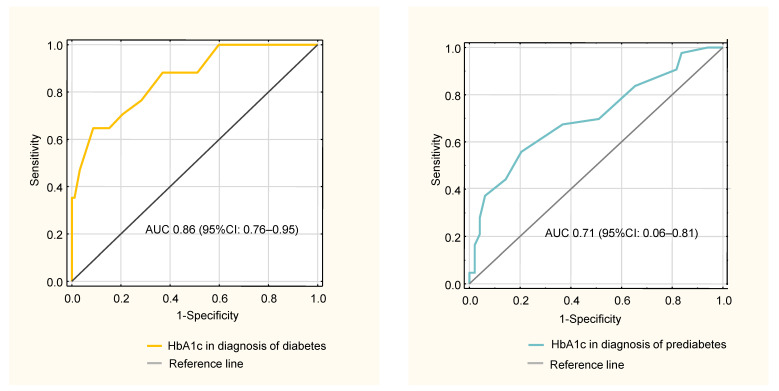
Receiver operating characteristic (ROC) curves comparing HbA1c and oral glucose tolerance test (OGTT) as diagnostic methods for diabetes and prediabetes in ASC patients. The OGTT result was used as the reference.

Receiver operating characteristic (ROC) curves comparing HbA1c and OGTT as diagnostic methods for diabetes and prediabetes in ASC patients are shown in Figure 3. 

## 4. Discussion

The results of our study indicate that HbA1c measurement is insufficient to detect diabetes in hospitalized patients with ACS. A complete OGTT performed shortly after discharge with FPG, and 2 h post-load values, seem to be necessary to adequately detect diabetes.

The OGTT performed shortly after discharge in ACS patients without known diabetes revealed diabetes in 15.6%, impaired glucose metabolism in 39.4%, and normoglycemia only in 45%. The presence of diabetes, suggested by an HbA1c ≥ 6.5% measured during the first two days of hospitalization, was much lower (9.1%), while prediabetes was much higher (51.2%) than the percentage indicated by OGTT. The results of our study confirm the important role of an OGTT in detecting previously undiagnosed diabetes. A study performed in the general population, using HbA1c, fasting glycemia and 2 h plasma glucose in OGTT, showed that, of those with diabetes, the vast majority—63%—were identified by 2 h OGTT glucose values alone [11]. In ACS patients, the OGTT was a much more sensitive diagnostic method for undiagnosed diabetes than HbA1c, fasting glycemia, or admission glycemia [17]. The much higher prevalence of diabetes and prediabetes in the last citied study—35% and 44%, respectively—was probably related to the fact that the study was performed in hyperglycemic ACS subjects with admission plasma glucose levels of 7.8–16 mmol/L.

The results of the study and findings of the authors citied above suggest that HbA1c, at the currently used cut-off point, has a low sensitivity in detecting undiagnosed diabetes in ACS patients. Screening and further diagnosis based on HbA1c values, without performing an OGTT, may result in significant underdiagnosis of diabetes in this group of patients. The diagnostic procedure for undiagnosed diabetes, relying mainly on HbA1c assay and its cut-off point of ≥6.5%, when performed on ACS patients, may miss 70% of undetected diabetes [17].

However, using the HbA1c method as a screening tool for diabetes is very easy and convenient in the course of acute illness, including ACS, the low sensitivity of the currently used cut-off point suggests the necessity of the OGTT test. It is worth debating whether OGTT should be performed in a majority of high-risk post-ACS patients, especially those with HbA1c > 5.7%, or whether it is necessary to search for another cut-off point for HbA1c diagnostic for diabetes in patients with cardiovascular diseases, including ACS.

The current cut-off HbA1c value diagnostic for diabetes (6.5%; 48 mmol/mL) was defined in a large study of 28,000 participants as the point at which at least moderate retinopathy, i.e., retinopathy that was more diabetes-specific, could be detected [4]. A recently published systematic review and meta-analysis of the diagnostic HbA1c cut-off point, based on microvascular complications, confirmed the high specificity of HbA1c values ≥6.5% in diagnosing diabetes; however, it was demonstrated that, at HbA1c < 6.5%, retinopathy increased at age >55 years, and an excess of microvascular complications (both retinopathy and nephropathy) may be present below the diabetes diagnostic threshold [19]. The authors of this study suggested that the sensitivity of this diagnostic method of type 2 diabetes may improve with a lower diagnostic cut-off point.

It may be supposed that the same phenomenon could exist in cardiovascular disorders. The results of our study suggest that the diagnostic diabetes threshold of HbA1c ≥ 6.5% is too high, and HbA1c levels of around 6.0% may be more suitable as a cut-off point for diabetes diagnosis in patients with ACS. A study suggesting a lower cut-off point of HbA1c for diagnosing undetected and preexisting diabetes in ACS patients was published over 15 years ago [20]. Diabetes was diagnosed by an OGTT test performed within 3 days of admission in the hospital; however, diabetes was arbitrarily classified as undiagnosed if HbA1c was ≥6.0%, or possible stress hyperglycemia with an HbA1c < 6.0%. OGTT performed 5–8 weeks later confirmed diabetes in all subjects with previous HbA1c ≥ 6.0%, and only in 16% of those with HbA1c < 6.0% [20]. The recently published meta-analysis assessing diagnostic accuracy of tests for type 2 diabetes suggest that at present recommended threshold of 6.5%, HbA1c is more specific and less sensitive in diagnosing diabetes in undiagnosed population in a community setting [21]. The optimal cut-off for diagnosing diabetes with HbA1c was estimated as 6.03% with a pooled sensitivity of 74% and specificity of 87%. Lowering the threshold for HbA1c to 6.03% for early detection of diabetes was suggested for consideration [21].

APG levels ≥ 200 mg/dL (11.1 mmol/L), which may be indicative of diabetes diagnosed at random conditions, were found in our study in only a very small number of subjects, and the mean APG values in patients with diabetes diagnosed post-discharge OGTT were much lower than the cut-off point indicated by diagnostic standards. APG levels in our ACS patients were, therefore, not useful to predict diabetes. Very similar data on the low predictable value of APG were shown for ACS patients with hyperglycemia [17]. Some studies suggest that the optimal cut-off points of glycemia diagnostic for diabetes in ACS patients, compared to the OGTT results, should be much lower than those that are currently used [22]. The optimal diagnostic cut-off point of admission glycemia compared to OGTT was found to be 7.7 mmol/L (138.6 mg/dL), with a sensitivity of 66% and specificity of 82% [22]. The same was reported for fasting glycemia levels of 5.8 mmol/L (104.4 mg/dL), with a sensitivity of 69% and specificity of 77% [22]. Our study demonstrates the possible presence of diabetes and prediabetes suggested by fasting glycemia measured within the first two days in the hospital. However, this was only a single FPG assay; the recorded values suggested almost the same percentage of diabetes, IFG, and normal glucose metabolism as the results of the complete OGTT performed after discharge. This may suggest that hyperglycemia detected in the course of ACS is a signal of undiagnosed and preexisting diabetes or prediabetes, rather than a transient stress abnormality in subjects with normal glucose metabolism. Due to this observation, fasting glycemia in the hospital seems to be a valuable screening method for diagnosing glucose abnormalities.

The results of our study and the findings of several other studies indicate the possibility of undiagnosed diabetes in patients with ACS syndrome, with HbA1c and/or admission glucose levels below the cut-off points used for diabetes diagnosis by the current standards of diabetes care. Not performing OGTT in an early but stable phase of the disease may result in a significant underdiagnosis of glucose abnormalities in this high-risk group.

The strength of our study is its very detailed characteristics, in terms of both the clinical and metabolic data of ACS patients participating in the study, which excluded coexisting comorbidities or abnormalities that might impact HbA1c levels. Another strength is the design of the study, in which we determine and compare HbA1c and OGTT diagnostic values, and assess other parameters, such as admission glycemia and fasting glycemia in the hospital. The time of the OGTT—2 weeks after discharge—is also very important. The test was performed in the quiet phase of the disease, which allowed the stress of hospitalization to be avoided, and the patients remained at home on their habitual diet. The time of the test prevented major changes in body mass or diet, which might occur in later weeks or months and might impact the glucose metabolism. 

The limitation of our study was the relatively small number of ACS patients diagnosed for glucose abnormalities, especially if different methods are compared. Although this number of participants is typical of this kind of study, the true prevalence of undiagnosed diabetes or impaired glucose metabolism should be confirmed in a larger cohort. The metabolic effect of the treatment with beta-blockers and statins initiated after ACS may rise some concern, but there was a very short period before performing OGTT, so their impact on glucose metabolism should not be substantial. 

## Figures and Tables

**Table 1:** Clinical characteristics of investigated subjects with acute coronary syndrome and no history of glucose abnormalities.

Parameter	Total Group *n* = 121	Women *n* = 33	Men *n* = 88	*p*
Age [years]	62 ± 12	61 ± 14	63 ± 11	ns
Body mass [kg]	84 ± 18	71 ± 12	85 ± 18	<0.02
BMI [kg/m^2^]	27.9 ± 5.2	28.9 ± 4.8	27.9 ± 5.2	ns
Systolic BP [mmHg]	127 ± 27	124 ± 17	128 ± 28	ns
Diastolic BP [mmHg]	78 ± 14	73 ± 9	78 ± 14	ns
STEMI	70%	82%	66%	ns
NSTEMI	30%	18%	34%	ns
Coronary artery disease				
1-artery	17.5%	33.3%	11.4%	ns
2-arteries	28.3%	27.3%	28.4%	ns
3-arteries	54.2%	36.4%	60.2%	<0.01
Hypertension	48%	30%	55%	ns
Dyslipidemia	52%	52%	52%	ns
History of myocardial infarction	13.2%	6.1%	15.9%	ns
History of invasive treatment (PCI, CABG)	8.3%	6.1%	9.1%	ns

Note: BMI—body mass index; BP—blood pressure; STEMI—ST-elevation myocardial infarction, NSTEMI—non-ST elevation myocardial infarction, PCI—percutaneous coronary intervention; CABG—coronary artery bypass graft; Statistical significance: ns—nonsignificant.

**Table 2:** Laboratory parameters of investigated subjects with acute coronary syndrome and no history of glucose abnormalities.

Parameter	Total Group *n* = 121	Women *n* = 33	Men *n* = 88	*p*
Chol-Tot. [mmol/L]	5.7 ± 1.7	6.0 ± 1.0	5.6 ± 1.8	ns
LDL [mmol/L]	3.6 ± 1.4	3.9 ± 1.0	3.5 ± 1.4	ns
HDL [mmol/L]	1.3 * (1.1; 1.5)	1.3 * (1.1; 1.5)	1.3 * (1.0; 1.5)	ns
TG [mmol/L]	1.7 ± 1.3	1.6 ± 0.7	1.7 ± 1.4	ns
Creatinine [μmol/L]	79.6 ± 22.1	67.2 ± 22.9	83.9 ± 22.1	<0.02
e-GFR [ml/min/1.73 m^2^]	85.7 ± 27.2	83.6 ± 35.2	86.4 ± 24.4	ns
Uric acid [mmol/L]	0.34 ± 0.10	0.32 ± 0.13	0.34 ± 0.09	ns
CRP [mmol/L]	3.62 * (1.39; 12.11)	4.29 * (2.55; 13.83)	3.3 * (1.24; 8.28)	ns
Na [mmol/L]	138 ± 3	138 ± 3	138 ± 3	ns
K [mmol/L]	4.2 ± 0.5	4.2 ± 0.4	4.2 ± 0.5	ns
RBC [×10^12^/L]	4.6 ± 0.4	4.5 ± 0.5	4.6 ± 0.5	ns
WBC [×10^9^/L]	10.5 ± 3.6	10.4 ± 3.7	10.6 ± 3.6	ns
HGB [mmol/L]	9.6 ± 7.6	8.5 ± 6.6	9.9 ± 8.6	<0.05
HCT [%]	41.8 ± 4.1	40.6 ± 4.4	42.3 ± 3.8	ns
MCV [fL]	88.9 ± 6.4	88.0 ± 4.3	89.2 ± 6.9	ns
PLT [×10^9^/L]	248 ± 79	277 ± 67	238 ± 81	ns

Note: Chol. Tot.—total cholesterol; LDL—low-density lipoprotein cholesterol; HDL—high-density lipoprotein cholesterol; TG—triglycerides, e-GFR—estimated glomerular filtration rate, CRP—C reactive protein, Na—sodium, K—potassium, RBC—red blood cells, WBC—white blood cells, HGB—hemoglobin, HCT—hematocrit, MCV—mean corpuscular volume of erythrocytes, PLT—blood platelets *—results presented as the median and quartiles. Statistical significance: ns—nonsignificant.

**Table 3:** HbA1c, admission and fasting glycemia in the hospital, and fasting and 2 h glycemia in OGTT.

Parameter	Total Group *n* = 121	Women *n* = 33	Men *n* = 88	*p*
HbA1c [%]	5.9 ± 0.8	5.9 ± 1.0	5.8 ± 0.6	ns
Admission glycemia [mmol/L]	7.0 ± 1.9	7.7 ± 2.6	6.7 ± 1.5	ns
FPG in the hospital [mmol/L]	5.9 ± 1.2	6.0 ± 1.1	5.9 ± 1.2	ns
FPG in OGTT * [mmol/L]	5.7 ± 1.1	5.6 ± 1.2	5.7 ± 1.1	ns
2-h PG in OGTT [mmol/L]	7.1 ± 2.0	6.7 ± 1.6	7.3 ± 2.2	ns

Note: HbA1c—glycated hemoglobin A1c, FPG—fasting glycemia, OGTT *—oral glucose tolerance test after discharge was performed in 109 patients (28 women and 81 men), Statistical significance: ns—nonsignificant.

**Table 4:** HbA1c, admission and fasting glycemia in the hospital in different glucose abnormalities diagnosed in OGTT performed two weeks after discharge.

OGTT Results *n* = 109	% (n)	HbA1c (%)	Admission Glycemia (mmol/L)	FPG in the Hospital (mmol/L)
Normal	45.0 (49)	5.5 ± 0.3	6.6 ± 1.4	5.5 ± 0.8
IFG	18.3 (20)	5.8 ± 0.3 ^a^	6.6 ± 1.6	5.8 ± 0.6 ^a^
IGT	21.1 (23)	5.8 ± 0.4 ^a^	6.7 ± 1.3	5.8 ± 1.2
DM	15.6 (17)	7.0 ± 1.8 ^bcd^	9.2 ± 3.1 ^bcd^	7.5 ± 1.4 ^bcd^

Note: OGTT—oral glucose tolerance test, HbA1c—glycated hemoglobin A1c, FPG—fasting plasma glucose, IFG—impaired fasting glucose, IGT—impaired glucose tolerance, DM—diabetes mellitus, N-normal glucose metabolism. ^a^ vs. N; *p* < 0.04; ^b^ vs. N; *p* < 0.01; ^c^ vs. IFG; *p* <0.01; ^d^ vs. IGT; *p* < 0.01.

**Table 5:** The prevalence of glucose abnormalities related to the applied diagnostics methods.

Laboratory Parameter	Normal Values	Pre-Diabetes	DM
HbA1c	39.7%	51.2%	9.1%
*n* = 121	48	62	11
FPG		IFG	
in the hospital	46.9%	35.7%	17.4%
*n* = 115	54	41	20
FPG		IFG	
in day of OGTT	54.1%	35.8%	10.1%
*n* = 109	59	39	11
FPG or 2-h glycemia		IFG+IGT	
in OGTT	45.0%	39.4%	15.6%
*n* = 109	49	43	17

Note: HbA1c—glycated hemoglobin A1c, FPG—fasting plasma glucose, OGTT—oral glucose tolerance test, IFG—impaired fasting glucose, IGT—impaired glucose tolerance, DM—diabetes mellitus.

**Table 6:** The classification of glucose abnormalities based on HbA1c levels and oral glucose tolerance test (OGTT) results.

OGTT	HbA1c		
	Normal	Prediabetes	DM
Normal 49	31	18	0
IFG 20	5	14	1
IGT 23	9	13	1
DM 17	2	8	7

Note: HbA1c—glycated hemoglobin A1c, OGTT—oral glucose tolerance test, IFG—impaired fasting glucose, IGT—impaired glucose tolerance, DM—diabetes.

## Data Availability

The data presented in this study will be made available from the authors upon reasonable request.
